# Application of Cas12j for *Streptomyces* Editing

**DOI:** 10.3390/biom14040486

**Published:** 2024-04-16

**Authors:** Lee Ling Tan, Elena Heng, Chung Yan Leong, Veronica Ng, Lay Kien Yang, Deborah Chwee San Seow, Lokanand Koduru, Yoganathan Kanagasundaram, Siew Bee Ng, Guangrong Peh, Yee Hwee Lim, Fong Tian Wong

**Affiliations:** 1 Institute of Molecular and Cell Biology (IMCB), Agency for Science, Technology and Research (A*STAR), 61 Biopolis Drive, Proteos #07-06, Singapore 138673, Singapore; tan_lee_ling@imcb.a-star.edu.sg (L.L.T.); elena_heng@imcb.a-star.edu.sg (E.H.);; 2 Singapore Institute of Food and Biotechnology Innovation (SIFBI), Agency for Science, Technology and Research (A*STAR), 31 Biopolis Way, Nanos #02-01, Singapore 138669, Singapore; leongcy@sifbi.a-star.edu.sg (C.Y.L.); ngwp@sifbi.a-star.edu.sg (V.N.); yanglk@sifbi.a-star.edu.sg (L.K.Y.); seowcs@sifbi.a-star.edu.sg (D.C.S.S.); yoganathank@sifbi.a-star.edu.sg (Y.K.); ngsb@sifbi.a-star.edu.sg (S.B.N.); 3 Institute of Sustainability for Chemicals, Energy and Environment (ISCE2), Agency for Science, Technology and Research (A*STAR), 8 Biomedical Grove, Neuros #07-01, Singapore 138665, Singapore; peh_guangrong@isce2.a-star.edu.sg (G.P.); lim_yee_hwee@isce2.a-star.edu.sg (Y.H.L.)

**Keywords:** CRISPR-Cas, Cas12j-2, *Streptomyces*, genome editing, natural product discovery

## Abstract

In recent years, CRISPR-Cas toolboxes for *Streptomyces* editing have rapidly accelerated natural product discovery and engineering. However, Cas efficiencies are oftentimes strain-dependent, and the commonly used *Streptococcus pyogenes* Cas9 (SpCas9) is notorious for having high levels of off-target toxicity effects. Thus, a variety of Cas proteins is required for greater flexibility of genetic manipulation within a wider range of *Streptomyces* strains. This study explored the first use of *Acidaminococcus* sp. Cas12j, a hypercompact Cas12 subfamily, for genome editing in *Streptomyces* and its potential in activating silent biosynthetic gene clusters (BGCs) to enhance natural product synthesis. While the editing efficiencies of Cas12j were not as high as previously reported efficiencies of Cas12a and Cas9, Cas12j exhibited higher transformation efficiencies compared to SpCas9. Furthermore, Cas12j demonstrated significantly improved editing efficiencies compared to Cas12a in activating BGCs in *Streptomyces* sp. A34053, a strain wherein both SpCas9 and Cas12a faced limitations in accessing the genome. Overall, this study expanded the repertoire of Cas proteins for genome editing in actinomycetes and highlighted not only the potential of recently characterized Cas12j in *Streptomyces* but also the importance of having an extensive genetic toolbox for improving the editing success of these beneficial microbes.

## 1. Introduction

Actinomycetes are highly productive factories of bioactive natural products (NPs) [1,2]. The activation and production of these pharmaceutically important secondary metabolites are typically triggered by environmental signals [3]. Unfortunately, under laboratory conditions, it is estimated that 80% of this chemical diversity cannot be observed [4,5,6]. To this end, various methods to activate these silent biosynthetic gene clusters (BGCs) have been investigated and deployed [7,8,9,10,11,12]. In our laboratory and others, CRISPR-Cas-mediated editing of actinomycetes, in particular *Streptomyces*, has been found to accelerate NP discovery and engineering [13,14,15,16].

**Figure 1:**
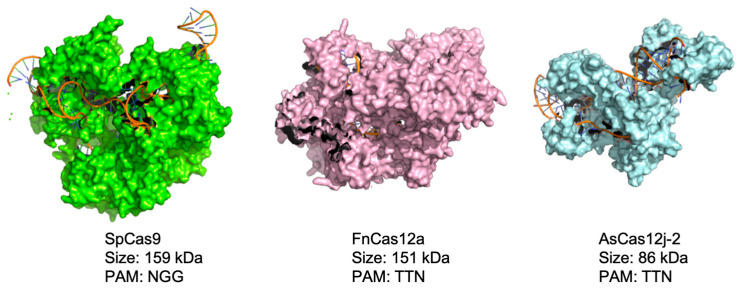
Representation of Cas proteins used in this study. Comparison of SpCas9 (PDB: 4OO8), FnCas12a (PDB: 6I1L), and AsCas12j-2 (PDB: 7LYS). Structures are shown to the same scale.

*Streptococcus pyogenes* Cas9 (SpCas9), *Staphylococcus aureus* Cas9 (SaCas9), *Streptococcus thermophilus* CRISPR 1 Cas9 (Sth1Cas9), and *Francisella tularensis* subsp. *novicida* U112’s type V-A Cas (FnCas12a, also known as FnCpf1) have been used for genome editing in *Streptomyces* [13,17]. Among these, Cas12a has been shown to be highly efficient (75–95%) for precise genome editing in the presence of a homology repair template in *Streptomyces* [18]. This efficiency is also comparable to that of the more commonly used SpCas9. In 2020, Pausch et al. reported the discovery and usage of the hypercompact Cas12 subfamily *Acidaminococcus* sp. Cas12j (AsCas12j), wherein the Cas protein has a single RuvC endonuclease domain for both the processing of crRNA and cleavage [19]. Although it has similar functions and capabilities to those of Cas12a, Cas12j is significantly smaller, being 757 amino acids (aa) long, as compared to SpCas9 (1368 aa) and FnCas12a (1300 aa) (Figure 1). This size advantage makes Cas12j an attractive candidate for the construction of plasmids and vector-based delivery into cells. The AsCas12j system was demonstrated to be active in vitro and in human and plant cells, and subsequently [19], we hypothesized that this would be functional in *Streptomyces.*

In this work, we aimed to further expand the Cas toolbox by characterizing the first examples of *Acidaminococcus* sp. Cas12j-mediated editing in *Streptomyces.* Although Cas12j-mediated editing did not achieve similar efficiencies in our model *Streptomyces* strains compared to prior studies, its transformation efficiencies were higher compared to those of SpCas9, suggesting less toxicity in *Streptomyces* for Cas12j. To further explore its transformation efficiencies and related toxicity, we used Cas12j for the upregulation of secondary metabolites via the activation of BGCs within *Streptomyces* sp. A34053, whose genome is largely inaccessible to the commonly used SpCas9. In this example, we also observed improved editing efficiencies with Cas12j compared to Cas12a (FnCas12a). This subsequently led to the upregulation of secondary metabolite production, increasing the accessibility of the metabolic space, which would otherwise not have been possible with either SpCas9 or Cas12a.

## 2. Materials and Methods

### 2.1. Construction of Genome-Editing Plasmids

To align with the codon usage of *Streptomyces*, the Cas12j-2 gene was codon-optimized and synthesized by Genscript, USA. DNA manipulations were conducted using OmniMAX^TM^ 2 (Thermo Fisher, Waltham, MA, USA). Plasmids used in this study and their corresponding Cas proteins are given in Table S1. Protospacers were initially incorporated through BbsI-mediated Golden Gate Assembly, followed by the introduction of the corresponding homology flanks via Gibson assembly, as described previously [20].

### 2.2. Target Site Prediction

PhytoCRISP-Ex software v.1.0 [21] was employed for target site prediction with potential sites defined as sites where seed regions did not completely match off-targets. Here, we specified seed regions as the last 15 bases of the guide RNA including PAM sequences.

### 2.3. Conjugation and Screening of Strains

Spore preparations, conjugation protocol, and the method for the screening of edits were adapted from Yeo et al., 2019 and Tan et al., 2022 [13,20]. To obtain the spores of *Streptomyces* sp. A34053, the strain was first propagated in SV2 media (15 g/L glucose, 15 g/L glycerol, 15 g/L soya peptone (Biobasic, Toronto, ON, Canada), and 1 g/L CaCO_3_, pH 7.0) and plated onto ISP Medium No. 4 (BD Biosciences, Franklin Lakes, NJ, USA). Spores were removed and resuspended in sterile TX buffer (50 mM Tris pH 7.4, 0.001% (*v*/*v*) Triton X). Intergeneric conjugation between *Streptomyces* sp. A34053 and DNA methylase-deficient WM3780 *Escherichia coli* donor cells was performed by transforming promoter knock-in constructs into WM3780. Apramycin selection was used to select for clones and screened via colony PCR. Successful PCR amplicons were screened for the presence of the promoter or promoters via restriction enzyme digestion. Positive samples were then validated with Sanger sequencing (Figures S2–S4). In this study, our target sample size was 8. However, we were limited by constraints such as exconjugant numbers or PCR challenges. Consequently, sample sizes for assessing genomic editing efficiencies ranged from n = 3 to 8.

### 2.4. Fermentation and Analysis

Wild-type *Streptomyces* sp. A34053 and mutated strains were cultured in 5 mL SV2 media for 3–5 days. Saturated seed cultures were diluted into fresh fermentation media in a 1:20 volume ratio and fermented under conditions of 200 rpm shaking at 30 °C in the dark. CA09LB (10 g/L beef extract, 4 g/L yeast extract, 20 g/L glucose, 3 g/L glycerol, pH 7.0) was used for fermentation. After 9 days, the cultures were pelleted and the separated biomass and supernatant were lyophilized. The dried samples were extracted using methanol and filtered through filter paper (Whatman Grade 4). The filtrates were reconstituted for analysis.

The extracts were analyzed on an Agilent 1290 Infinity LC System coupled to an Agilent 6540 accurate-mass quadrupole time-of-flight (QTOF) mass spectrometer. A quantity of 5 µL of extract was injected onto a Waters Acquity UPLC BEH C18 column (2.1 × 50 mm, 1.7 µm). Mobile phases were water (A) and acetonitrile (B), both with 0.1% formic acid. The analysis was performed at a flow rate of 0.5 mL/min under gradient elution of 2% B to 100% B in 8 min. MS data was acquired in positive electrospray ionization (ESI) mode. The typical QTOF operating parameters were as follows: sheath gas nitrogen, 12 L/min at 325 °C; drying gas nitrogen flow, 12 L/min at 350 °C; nebulizer pressure, 50 psi; nozzle voltage, 1.5 kV; and capillary voltage, 4 kV. Lock masses in positive ion mode were as follows: purine ion at m/z 121.0509 and HP-0921 ion at m/z 922.0098.

## 3. Results

### 3.1. Design and Characterization of an AsCas12j-2 Functional Vector

Out of the three AsCas12j proteins examined previously [19], AsCas12j-2 was reported to be self-processing and have high levels of editing efficiencies. It was subsequently chosen to represent the Cas12j family in this study. Here, to characterize AsCas12j-2, we examine its transformation and genomic editing efficiencies against Cas9 and FnCas12a [13]. The Cas proteins in this study are based on an all-in-one pCRISPomyces-2 plasmid (Addgene #61737 [22]), which consists of the respective Cas protein and sgRNA/cRNA under the two distinct promoters. These two promoters are consistent throughout the plasmids.

To prepare AsCas12j-2 for its utilization in *Streptomyces*, we replaced the Cas protein in the pCRISPomyces-2 plasmid (Addgene #61737 [22]) with codon-optimized AsCas12j-2. The corresponding crRNA sequence “5-GTCGGAACGCTCAACGATTGCCCCTCACGAGGGGAC-3” was inserted after the promoter transcribing the guide RNA [19]. The general annotation of the elements in this final plasmid is shown in Figure S1. Final editing constructs were constructed by inserting homology repair templates and guide RNAs (Table 1, Figure S2).

Even though it is an efficient tool in genome editing for some *Streptomyces* strains, SpCas9′s high toxicity levels often resulted in no exconjugants in previous studies [13]. To compare the levels of toxicity of AsCas12j-2, we transformed it into *Streptomyces* strains that we had previously tested for Cas editing [13]. Transformation efficiencies for the AsCas12j-2 plasmid in the absence of a guide RNA or homology repair template yielded >400 exconjugants in *Streptomyces* sp. NRRL S-244. This contrasts with 0 exconjugants in our prior study [13] for SpCas9. Additional investigation of its transformation efficiencies with other genetically inaccessible strains also exhibited a similar trend wherein AsCas12j-2 yielded higher number of exconjugants against Cas9 proteins (Table S1). Nonetheless, further characterization suggests that its editing efficiencies are generally lower compared to those of other Cas systems (Table 1). The finding that a higher proportion of microbes carrying AsCas12j-2 may not undergo editing could be contributed to its lower toxicity levels and high transformation efficiencies within *Streptomyces.*

### 3.2. Editing in a Previously Inaccessible Strain

**Figure 2:**
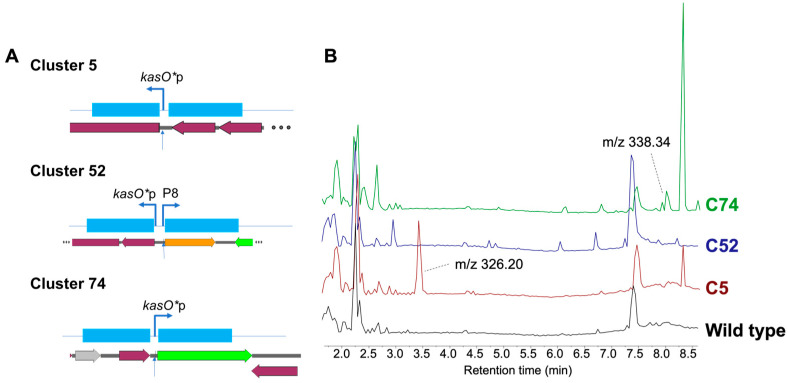
(**A**) Schematic homology flanks used for *Streptomyces* sp. A34053 editing along with (**B**) heat map representation of secondary metabolite production in wild-type and edited mutants for Cluster 5, 52, and 74 (Table 2). (**A**) Homology flanks (blue) with insertion of promoters are designed as the figures, where each flank is ~2 kb in length and 97 bps (kasO*p) or 778 bps (kasO*p with p8 promoter) is inserted at the specified sites. The sites of the protospacer are also indicated on the genome with arrows. Legend of genes on the genome annotation—grey: domain of unknown function; orange: transporter; green: regulator; red: biosynthetic enzymes. (**B**) Representative LCMS spectrum comparison between extracts of mutants and wild type. RT: retention time (min). MS spectra are shown in Figures S6 and S7.

To demonstrate the utility of Cas12, we selected a strain arising from the A*STAR Natural Organism Library (NOL, [23]) *Streptomyces* sp. A34053, which has 99.8% 16S sequence identity in relation to *Streptomyces luridiscabiei*. Earlier genome-based discovery, via AntiSMASH [24], revealed three unique gene clusters with low homology to known BGCs, including a hybrid Type I polyketide- non ribosomal peptide synthetase (NRPS), NRPS, and Type I polyketide (annotated as Clusters 5, 52, and 74; Figure 2A). These three different clusters (Figure 2A) were targeted for promoter exchange to enable cluster activation [25]. Cluster activation strategies include targeting the overexpression of a regulator or open reading frame of the main biosynthetic enzymes via the insertion of strong constitutive promoters, such as kasO*p [26] and p8 [25]. However, conducting initial precise editing studies has been challenging due to the inability to introduce the SpCas9-containing plasmid (Addgene #61737) into the strain. Conjugation with this plasmid, lacking both protospacer and homology repair templates, using our current protocols [13,20] has thus far failed to yield any transformants. As a result, we considered *Streptomyces* sp. A34053 as an ideal opportunity to evaluate the Cas12 systems for genome editing. Since FnCas12a and AsCas12j-2 belong to the Type V CRISPR-Cas system and recognize similar PAM sites (TTN) and FnCas12a was observed to be successful in previous experiments [13], we also compared the genome editing capabilities of AsCas12j-2 with FnCas12a.

The same protospacer (24 bps) and homology flanks were used for both Cas proteins, FnCas12a and AsCas12j-2, since they use the same PAM site. In the editing of all three clusters, AsCas12j-2 was functional and was able to insert the new promoter (Table 2, Figures S3–S5). Interestingly, both AsCas12j-2 and FnCas12a gave similar numbers of exconjugants, but AsCas12j-2 had a consistently higher rate of edits whilst FnCas12a yielded no edited strains (Table 2). This suggests that although AsCas12j-2 and FnCas12a have similar toxicity levels within *Streptomyces* sp. A34053, AsCas12j-2 outperforms FnCas12a in genome editing within the context of these clusters and this strain. Although metabolite profiles are influenced by a myriad of factors encompassing both global and localized regulation within the organism, with the promoter exchange, the production of distinct metabolites was observed for edited mutants for C74 (338.34 m/z) and C5 (326.20 m/z) as compared to the wild-type strain. However in C52 edited mutants, where a bidirectional promoter was inserted, despite changes to the metabolite production profiles from the wild type, there was no significant production of a new compound (Figure 2B).

## 4. Discussion

To harness the full biosynthetic potential of *Streptomyces*, much effort has been placed in expanding the Cas toolbox for the precise engineering of biosynthetic pathways within these organisms. The Cas protein, its size, its associated levels of toxicity, its PAM recognition sites, and its efficiency in editing the genome are all important factors for consideration in its application in *Streptomyces* [27]. In this study, we successfully applied the hypercompact Cas12j for *Streptomyces* usage and showed that the editing efficiencies varied depending on the species. Specifically, we observed that AsCas12j-2 exhibited lower editing efficiency compared to FnCas12a in *Streptomyces albus* J1074 and *Streptomyces* sp. NRRL S-244. However, these efficiencies are highly strain-dependent; in *Streptomyces* sp. A34053, among FnCas12a and AsCas12j-2, only AsCas12j-2 had the ability to perform genetic editing. These findings highlight the importance of having an expanded Cas toolbox to accommodate the diverse genome editing requirements in different bacterial strains.

Being almost half the size of the Cas9 family of proteins and less toxic as compared to SpCas9 or FnCas12a, we believe that the hypercompact AsCas12j-2 will be a valuable tool in the strategy to generate genomic perturbations in *Streptomyces*. Previous studies have also shown successful applications of various CRISPR-Cas strategies from *Streptomyces* to the rare Actinomycetes without modifications or the addition of helper proteins [27,28,29,30,31,32]. In the same manner, we predict that the usage of Cas12j-2 could also be applied across the Actinomycetes family.

## Figures and Tables

**Table 1:** Editing efficiencies and exconjugant outputs of AsCas12j-2.

Cas Constructs ^a^	Editing Efficiency ^b^	# Exconjugants ^c^	References
***Streptomyces albus* J1074**			
**AsCas12j-2 (template 2)**	**12.5% (1/8)**	**>400**	**This study**
FnCas12a (template 2)	87% (7/8)	>400	[13]
SpCas9 (template 1)	100% (8/8)	>400	[13]
SaCas9 (template 1)	87% (7/8)	>400	[13]
Sth1Cas9 (template 2)	100% (8/8)	>400	[13]
***Streptomyces* sp. NRRL S-244**			
**AsCas12j-2 (template 1)**	**62.5% (5/8)**	**>400**	**This study**
FnCas12a (template 1)	100% (8/8)	38	[13]

^a^ Schematics of templates are given in Figure S2. ^b^ Editing efficiency, given by the number of correct clones validated through Sanger sequencing over the total number of clones picked. ^c^ Number of exconjugants observed per 100 µL of spore preparation used in each conjugation. A typical spore prep contains ~10^6^–10^7^ spores/mL, as determined via serial dilution plating. To compare AsCas12j-2 against other Cas proteins, we have included previous data as referenced from [13].

**Table 2:** Editing efficiencies of AsCas12j-2 and FnCas12a in *Streptomyces* sp. A34053.

Cluster	Cas Constructs	Insertion (kb)	Editing Efficiency ^a^	# Exconjugants ^b^
5	AsCas12j-2	0.1	50% (3/6)	>50
	FnCas12a		0% (0/8)	>50
74	AsCas12j-2	0.1	75% (3/4)	4
	FnCas12a		0% (0/6)	6
52 ^c^	AsCas12j-2	1	75% (3/4)	7

^a^ Editing efficiency, given by the number of correct clones validated using Sanger sequencing over the total number of clones picked. ^b^ Number of exconjugants observed per 100 µL of spore preparation used in each conjugation. A typical spore prep contains ~10^6^–10^7^ spores/mL as determined via serial dilution plating. ^c^ FnCas12a was not used for this cluster due to difficulties in assembly of the editing construct.

## Data Availability

The Cas12j-2 plasmid (Figure S1) is available in Addgene (plasmid # 191655).

## Supplementary Materials

The following supporting information can be downloaded at: https://www.mdpi.com/article/10.3390/biom14040486/s1, Figure S1: General map of all-in-one editing CRISPR-Cas construct for one-step genome editing of *Streptomyces* using AsCas12j-2; Figure S2: Schematics of target sequences and homology arms for *Streptomyces albus* & *Streptomyces* sp. NRRLS-244; Figure S3: Representative trace of edited genome sequence (insertion of *kasO**p-Grey) for cluster 5 (ctg1_566-Red) in *Streptomyces* sp. A34053; Figure S4: Representative trace of edited genome sequence (insertion of P8-Grey) for cluster 52 (ctg2_2844-Red) in *Streptomyces* sp. A34053 & (insertion of *kasO**p-Grey) for cluster 52 (ctg2_2843-Red) in *Streptomyces* sp. A34053; Figure S5: Representative trace of edited genome sequence (insertion of *kasO**p-Grey) for cluster 74 (ctg4_545-Red) in *Streptomyces* sp. A34053; Figure S6: MS1 Spectra of retention time 3.41 min (Figure 2); Figure S7: MS1 Spectra of retention time 7.96 min (Figure 2); Table S1: Exconjugant outputs with the all-in-one pCRISPomyces-2 plasmids encoding for different Cas proteins. References [9,33] are cited in the supplementary materials.
