# Supplementary material for: Application of Cas12j for Streptomyces Editing
Source: Biomolecules. 2024 Apr 16;14(4):486. doi: 10.3390/biom14040486 (PMC11048056; doi:10.3390/biom14040486)
Supplement: Supplementary file 1 [file biomolecules-14-00486-s001.zip › biomolecules-2818405-supplementary.pdf]

Communication

Application of Cas12j for Streptomyces Editing

Lee Ling Tan [1,†], Elena Heng [1,†], Chung Yan Leong [2], Veronica Ng [2,] Lay Kien Yang [2], Deborah Chwee San Seow [2], Lokanand Koduru [1], Yoganathan Kanagasundaram [2], Siew Bee Ng [2], Guangrong Peh [3], Yee Hwee Lim [3] and Fong Tian Wong [1,3,*]

[1] Institute of Molecular and Cell Biology (IMCB), Agency for Science, Technology and Research (A*STAR), 61 Biopolis Drive, Proteos #07-06, Singapore 138673, Singapore; tan_lee_ling@imcb.a-star.edu.sg (L.L.T.); elena_heng@imcb.a-star.edu.sg (E.H.)

[2] Singapore Institute of Food and Biotechnology Innovation (SIFBI), Agency for Science, Technology and Research (A*STAR), 31 Biopolis Way, Nanos #02-01, Singapore 138669, Singapore; leongcy@sifbi.a-star.edu.sg (C.Y.L.); ngwp@sifbi.a-star.edu.sg (V.N.); yanglk@sifbi.a-star.edu.sg (L.K.Y.); seowcs@sifbi.a-star.edu.sg (D.C.S.S.); yoganathank@sifbi.a-star.edu.sg (Y.K.); ngsb@sifbi.a-star.edu.sg (S.B.N.)

[3] Institute of Sustainability for Chemicals, Energy and Environment (ISCE2), Agency for Science, Technology and Research (A*STAR), 8 Biomedical Grove, Neuros #07-01, Singapore 138665, Singapore; peh_guangrong@isce2.a-star.edu.sg (G.P.); lim_yee_hwee@isce2.a-star.edu.sg (Y.H.L.)

* Correspondence: wongft@imcb.a-star.edu.sg

† These authors contributed equally to this work.

Supplementary Material

Figure S1. General map of all-in-one editing CRISPR-Cas construct for one-step genome editing of *Streptomyces* using AsCas12j-2

Figure S2. Schematics of target sequences and homology arms for *Streptomyces albus* & *Streptomyces* sp. NRRLS-244.

Figure S3. Representative trace of edited genome sequence (insertion of *kasO**p-Grey) for cluster 5 (ctg1_566-Red) in *Streptomyces* sp. A34053.

Figure S4. Representative trace of edited genome sequence (insertion of P8-Grey) for cluster 52 (ctg2_2844-Red) in *Streptomyces* sp. A34053 & (insertion of *kasO**p -Grey) for cluster 52 (ctg2_2843-Red) in *Streptomyces* sp. A34053.

Figure S5. Representative trace of edited genome sequence (insertion of *kasO**p-Grey) for cluster 74 (ctg4_545-Red) in *Streptomyces* sp. A34053.

Figure S6. MS1 Spectra of retention time 3.41 min (Figure 2).

Figure S7. MS1 Spectra of retention time 7.96 min (Figure 2).

Table S1. Exconjugant outputs with the all-in-one pCRISPomyces-2 plasmids encoding for different Cas proteins.

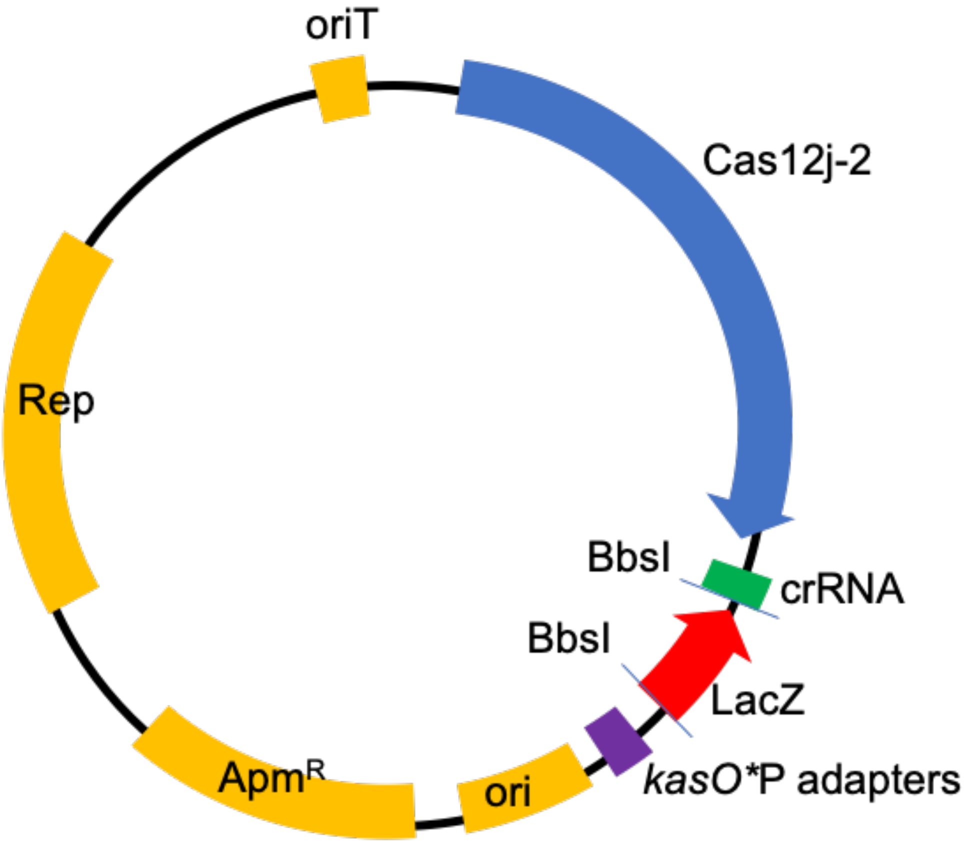

Figure S1. General map of all-in-one editing CRISPR-Cas construct for one-step genome editing of *Streptomyces* using AsCas12j-2. Rep: Replicon, ApmR: aparamycin resistance cassette, ori: origin of replication, oriT: origin of transfer, LacZ: LacZ operon for screening, *kasO**p adapters for additional of homology flanks, crRNA: CRISPR-RNA, BbsI sites for golden gate assembly of protospacer.

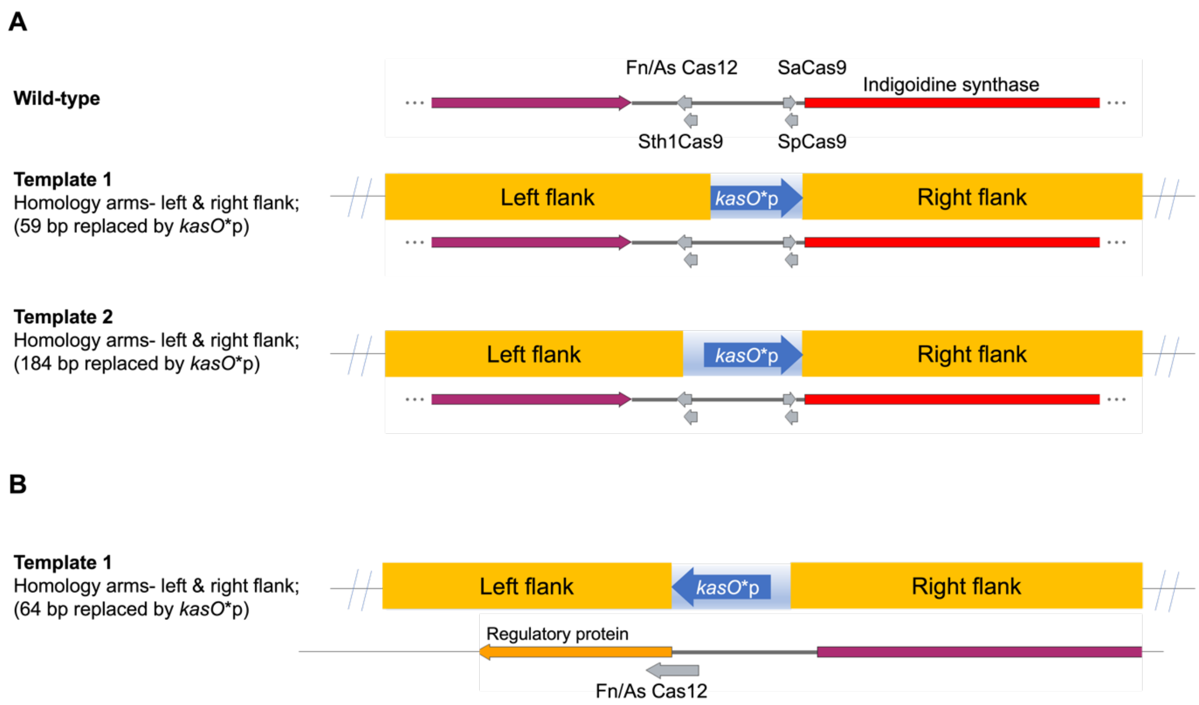

Figure S2. Schematics of target sequences and homology arms for Table 1.

(A) Schematics illustrating the protospacers (depicted as grey arrows) and homology arms (highlighted in yellow) for various Cas proteins in *Streptomyces albus* (Table 1), aimed at integrating *kasO** promoter (*kasO**p) before indigoidine synthase. The intergenic region spanning between the two genes measures 280 bps. The design of the homology arms within the editing templates is to minimize any disturbance to the native genome structure.

(B) Schematics of the Cas12 protospacer and corresponding homology arms intended for the gene editing in *Streptomyces* sp. NRRLS-244 (Table 1). The position of the target sequence for the specified Cas12 proteins are annotated (grey arrow). The homology arms within the editing templates (depicted in solid yellow) are also annotated.

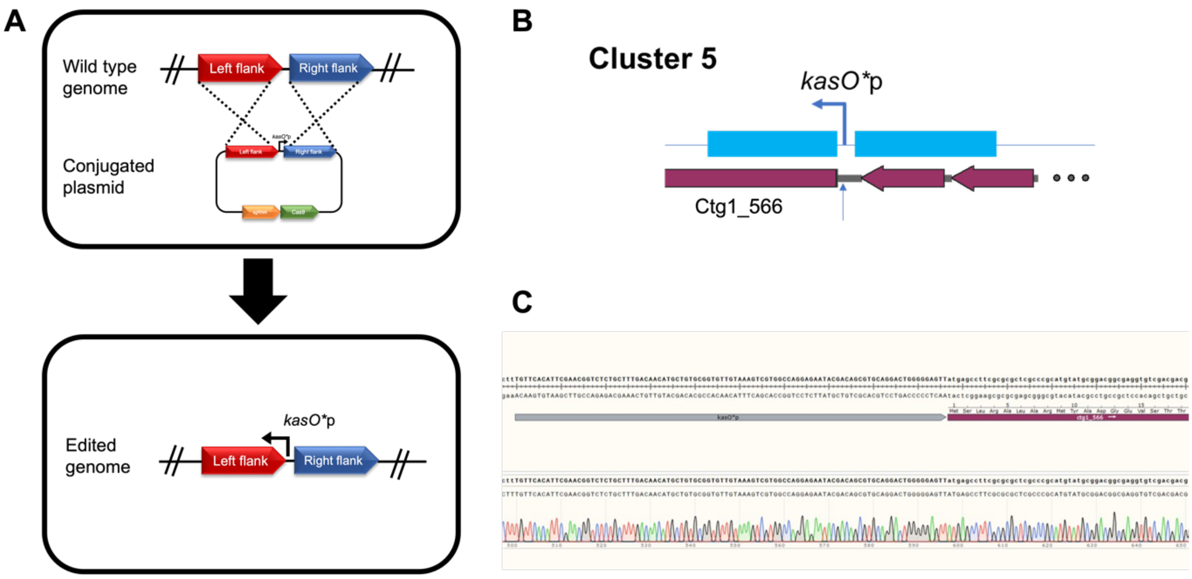

Figure S3. (A) Schematics for CRISPR-Cas mediated editing. (B) Schematics of homology arms for insertion. (C) Representative trace of edited genome sequence (insertion of *kasO**p-Grey) for cluster 5 (ctg1_566-Red) in *Streptomyces* sp. A34053.

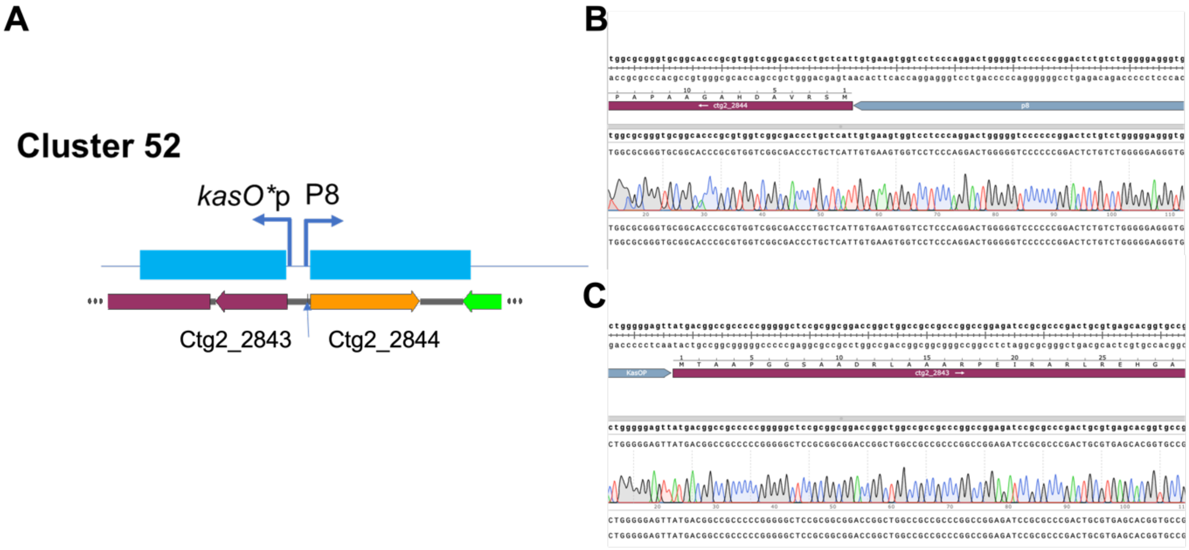

Figure S4. (A) Schematics of homology arms for insertion. (B) Representative trace of edited genome sequence (insertion of P8-Grey) for cluster 52 (ctg2_2844-Red) in *Streptomyces* sp. A34053. (C) (insertion of *kasO**p -Grey) for cluster 52 (ctg2_2843-Red) in *Streptomyces* sp. A34053.

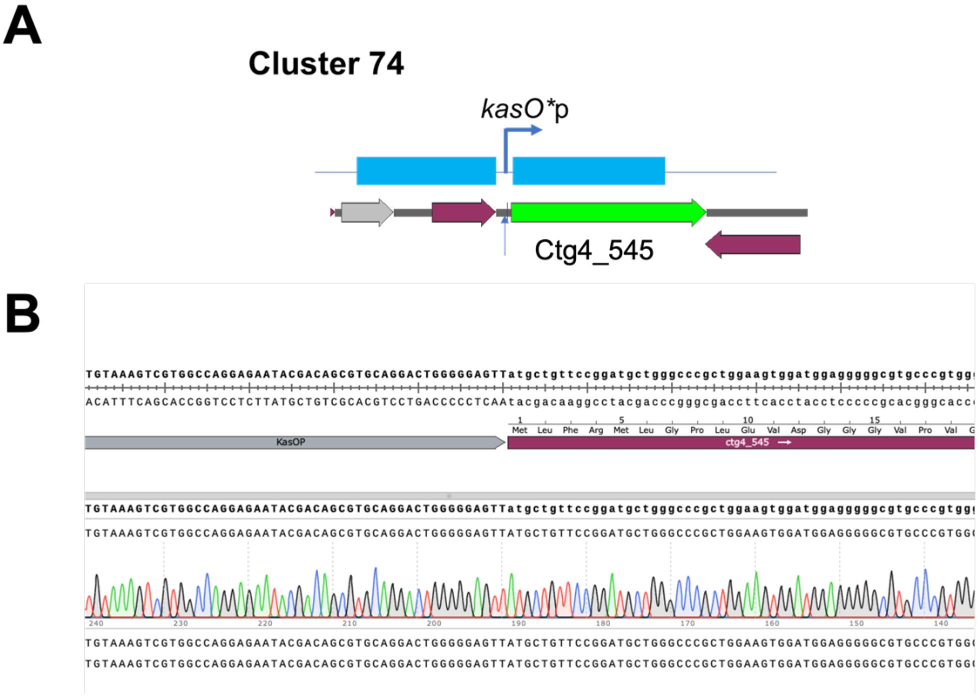

Figure S5. Representative trace of edited genome sequence (insertion of *kasO**p-Grey) for cluster 74 (ctg4_545-Red) in *Streptomyces* sp. A34053.

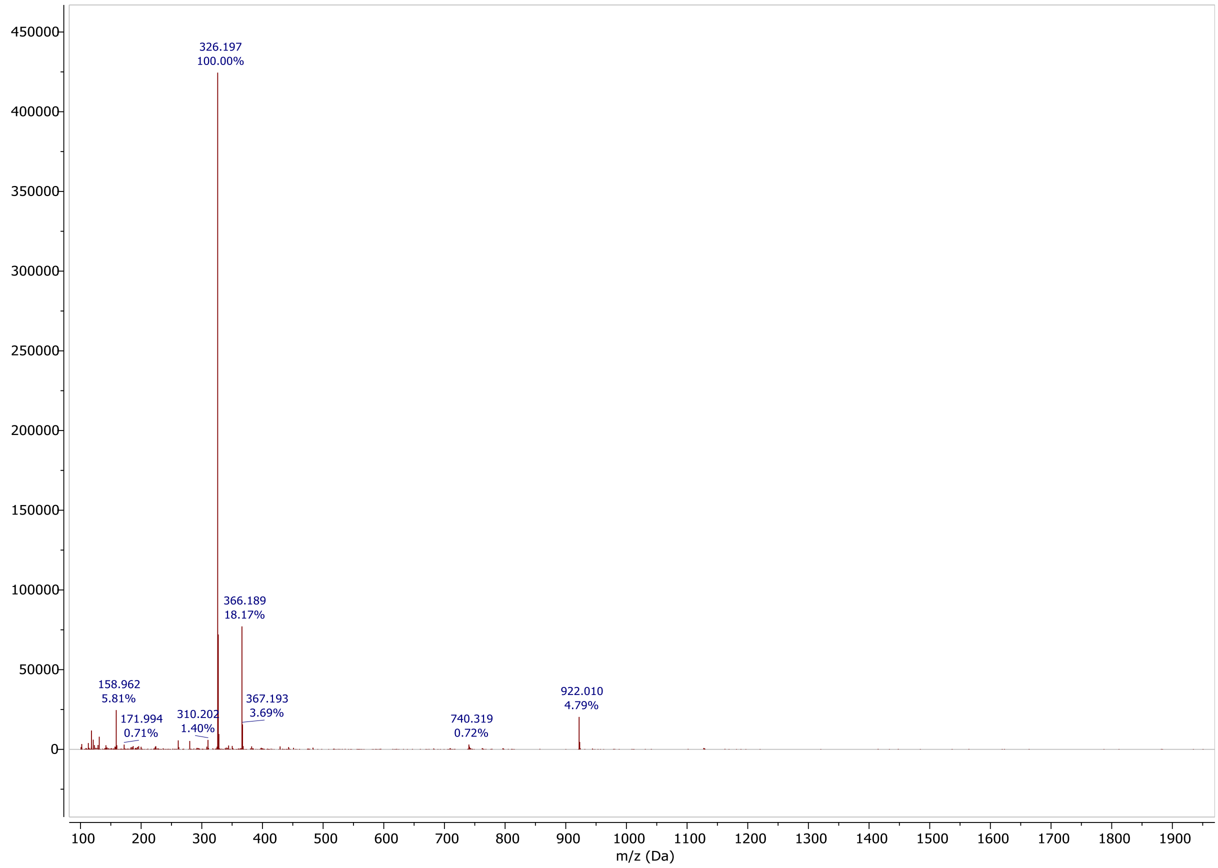

Figure S6. MS1 Spectra of retention time 3.41 min from *Streptomyces* sp. A34053-Cluster 5 edited mutant's LC-MS indicating m/z 326.20 as base peak (Figure 2).

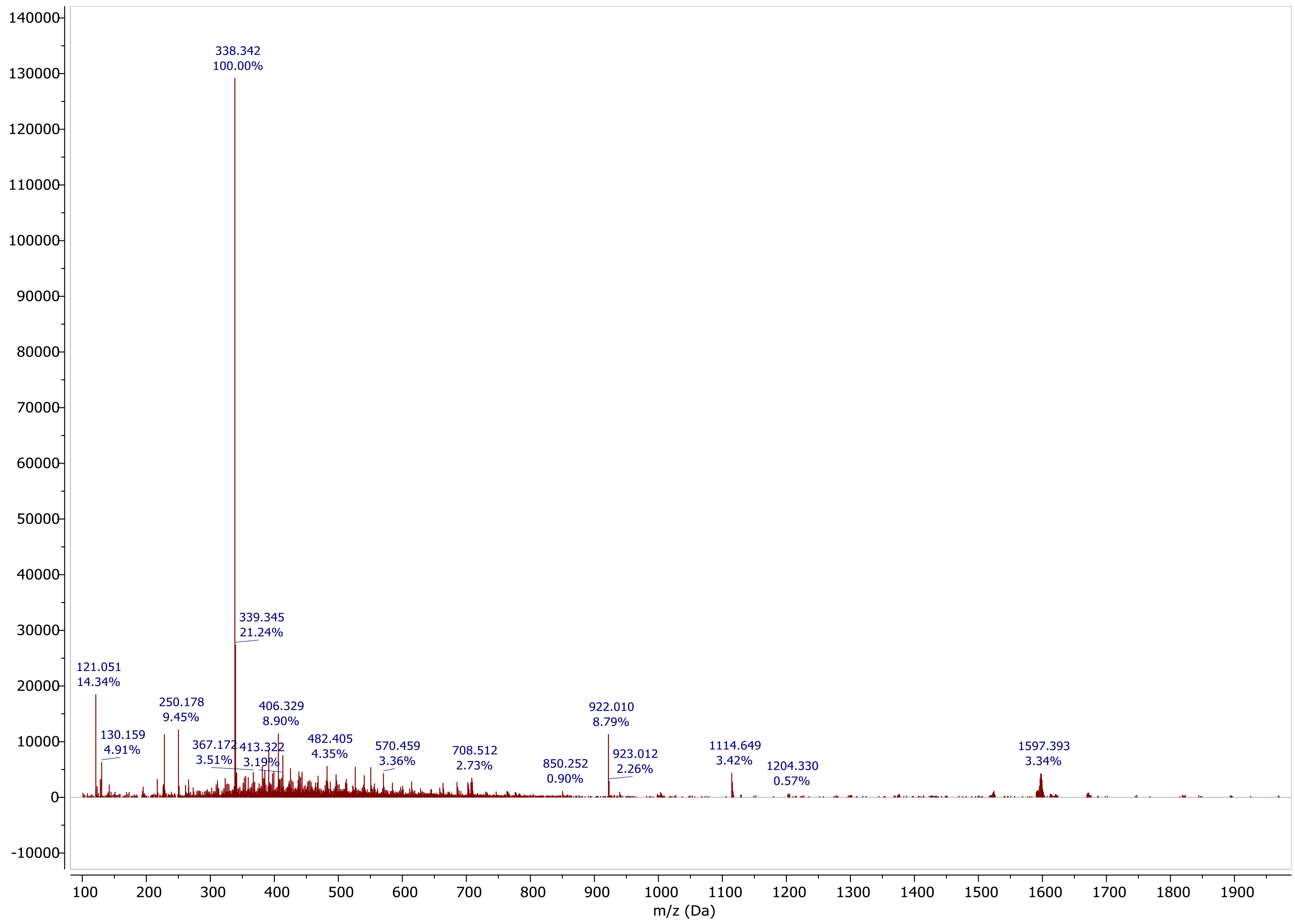

Figure S7. MS1 Spectra of retention time 7.96 min from *Streptomyces* sp. A34053-Cluster 74 edited mutant's LC-MS indicating m/z 338.34 as base peak (Figure 2).

Table S1. Exconjugant outputs with the all-in-one pCRISPomyces-2 plasmids encoding for different Cas proteins. Transformation was performed with the plasmids containing Cas proteins only; no protospacers or homology arms were inserted into these plasmids.

Plasmid (Addgene#)	Cas protein	Number of exconjugants observed[1]				
		Strains				
		A5252[2]	*S. lividans*	A8567[3]	A8274[4]	A793[5]
129553	SaCas9	0	3	4	0	0
61737	SpCas9	0	3	0	0	0
129552	Sth1Cas9	0	4	0	0	0
129554	FnCas12a	6	6	10	0	0
191655	AsCas12j-2	2	24	8	1	0

[1]Number of exconjugants observed per 20 μL of spore preparation used in each conjugation. A typical spore prep contains ~10^6–10^7 spores/mL as determined by serial dilution plating.

[2] From rRNA blast results of 16S, A5252 is similar to *Streptomyces aldersoniae*, 100% (1).

[3] From rRNA blast results of 16S, A8567 is similar to *Streptomyces abikoensis*, 99.87% (1).

[4] From rRNA blast results of 16S, A8274 is similar to *Micromonospora oryzae*, 99.77% (1).

[5] (2)

References:

1. Tay, D.W.P.; Tan, L.L.; Heng, E.; Zulkarnain, N.; Ching, K.C.; Wibowo, M.; Chin, E.J.; Tan, Z.Y.Q.; Leong, C.Y.; Ng, V.W.P.; et al. Exploring a General Multi-Pronged Activation Strategy for Natural Product Discovery in Actinomycetes. Commun Biol 2024, 7, 50,

2. Heng E, Lim YW, Leong CY, Ng VWP, Ng SB, Lim YH, et al. Enhancing armeniaspirols production through multi-level engineering of a native Streptomyces producer. Microb Cell Fact. 2023;22(1).
